# The role of microRNAs in the osteogenic and chondrogenic differentiation of mesenchymal stem cells and bone pathologies

**DOI:** 10.7150/thno.55664

**Published:** 2021-04-30

**Authors:** Maria Rosa Iaquinta, Carmen Lanzillotti, Chiara Mazziotta, Ilaria Bononi, Francesca Frontini, Elisa Mazzoni, Lucia Oton-Gonzalez, John Charles Rotondo, Elena Torreggiani, Mauro Tognon, Fernanda Martini

**Affiliations:** Department of Medical Sciences, Section of Experimental Medicine, School of Medicine, University of Ferrara. Ferrara, Italy.

**Keywords:** epigenetics, microRNAs, MSC differentiation, disease, tumour

## Abstract

Mesenchymal stem cells (MSCs) have been identified in many adult tissues. MSCs can regenerate through cell division or differentiate into adipocytes, osteoblasts and chondrocytes. As a result, MSCs have become an important source of cells in tissue engineering and regenerative medicine for bone tissue and cartilage. Several epigenetic factors are believed to play a role in MSCs differentiation. Among these, microRNA (miRNA) regulation is involved in the fine modulation of gene expression during osteogenic/chondrogenic differentiation. It has been reported that miRNAs are involved in bone homeostasis by modulating osteoblast gene expression. In addition, countless evidence has demonstrated that miRNAs dysregulation is involved in the development of osteoporosis and bone fractures. The deregulation of miRNAs expression has also been associated with several malignancies including bone cancer. In this context, bone-associated circulating miRNAs may be useful biomarkers for determining the predisposition, onset and development of osteoporosis, as well as in clinical applications to improve the diagnosis, follow-up and treatment of cancer and metastases. Overall, this review will provide an overview of how miRNAs activities participate in osteogenic/chondrogenic differentiation, while addressing the role of miRNA regulatory effects on target genes. Finally, the role of miRNAs in pathologies and therapies will be presented.

## Introduction

Mesenchymal stem cells (MSCs) are non-hematopoietic multipotent cells and, as a result of their potential promising properties, are widely studied in regenerative medicine 1. MSCs can renew themselves through cell division, whereas they differentiate both into osteoblasts and chondrocytes after exposure to specific soluble factors in the microenvironment. *In vitro*, osteogenic differentiation of stem cells typically involves the use of dexamethasone, β-glycerolphosphate and ascorbic acid 2 while MSCs placed in aggregate in a medium containing specific component, such as dexamethasone, ascorbate-2-phosphate, selenious acid, sodium pyruvate and transforming growth factor β (TGF-β) will undergo chondrogenic differentiation 3.

**Figure 1:**
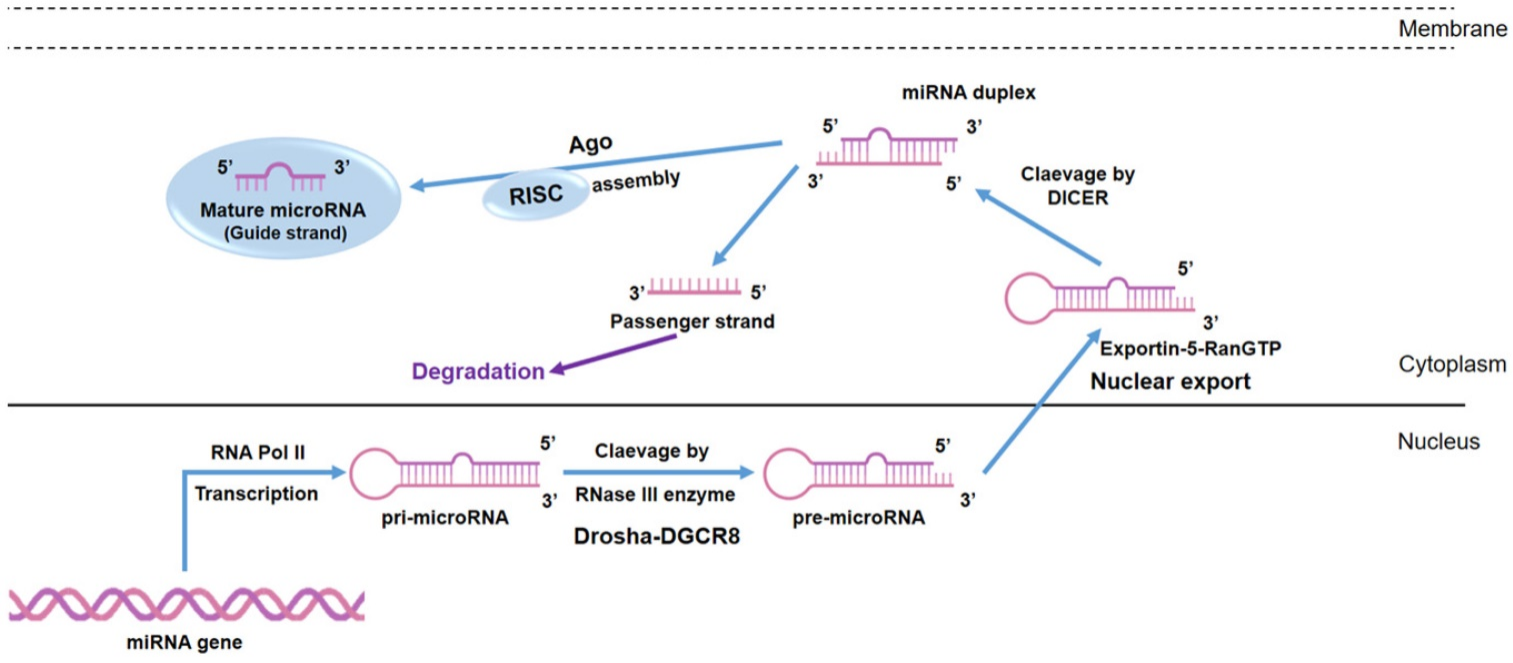
** MiRNA biogenesis.** MiRNAs are transcribed by RNA polymerases II (Pol II) in the form of a first precursor called primary miRNA (pri-miRNA). The pri-miRNA is converted into the precursor miRNA (pre-miRNA) via the cutting activity of the Drosha enzyme, a nuclear endoribonuclease III. Pre-miRNAs are exported into the cytoplasm as a result of the action of Ran-GTP and Exportin-5, a nuclear export factor. In the cytoplasm, Dicer clivates the pre-miRNA into a double stranded miRNA (miRNA duplex) of about 18-22 nt. The mature miRNA strand is incorporated into the RNA- induced silencing complex (RISC), which guides the miRNAs to the 3′UTR of its target.

Because several epigenetic factors play an important role in MSCs differentiation, a large number of studies have been conducted on the biological properties of MSCs and factors that might facilitate MSC chondrogenic/osteogenic differentiation as well as scaffolds/biomaterials for tissue engineering 4,5. Indeed, biomaterials provide the biological structure for MSCs to stimulate chondrogenic 6 and osteogenic 7,8 differentiation. The addition of ions 9 has been proved to enhance the osteogenic potential of scaffolds. On the other hand, biological macromolecules such as growth factors 10 and microRNAs (miRNAs) can be associated with scaffolds in order to increase differentiation potential 11. MiRNAs represent a family of small single-stranded, non-coding RNA molecules consisting of approximately 21-25 nucleotides (nt) which were discovered in nematode *Caenorhabditis elegans* and they were considered small temporal RNAs 12. It has been predicted that miRNAs account for 1-5% of the human genome, regulate at least 30% of protein-coding genes 13 and are known to be generated through a multi-step process (Figure 1). Moreover, it is known that miRNAs play a pivotal role in musculoskeletal functions, such as cell differentiation, survivability and apoptosis. To date, the most well-known function of miRNAs is the ability to negatively regulate gene expression at post-transcriptional level by interfering with target messenger RNA (mRNA) translation 14. The degree and nature of complementarity between the miRNA seed site and the 3′-UTR of its target mRNA determined the gene silencing mechanism: if complementarity is complete, the mRNA target will be degraded; if complementarity is partial, mRNA target translation is inhibited 14 causing a reduction of protein levels. Several computational algorithms have been developed in order to predict miRNA targets. These include miRWalk, Diana-Tools, miRanda, Microrna.org, mirDB, TargetMiner, TargetScan and PicTar. This software is able to predict miRNA targets based on bioinformatic tools, although, each target has to be experimentally validated using different techniques such as PCR and Western Blot after prediction.

MiRNAs are endogenous oligonucleotides, which can be released into the extracellular space and therefore are found in almost all body 15 and exhibit great potential as biomarkers for non-invasive cancer diagnosis and prognosis 16, as well as for therapy and surgical treatment responses 16. Targeted drugs based on molecules which mimic the action of miRNAs, or molecules which contrast its action (known as antago-miR), have been developed to cure different diseases, including cancer 17.

In addition, mimic or antago-miR have been proposed not only for therapy monitoring, but also as targets 18 for innovative therapeutic approaches in regenerative medicine in association with stem cells 5 and scaffolds 19 in order to improve tissue regeneration. MiRNAs could positively or negatively regulate chondrogenic and osteogenic differentiation by targeting either negative or positive genes or transcription factors (TFs), respectively. However, some miRNAs exert both stimulatory and inhibitory effects during chondrogenic and osteogenic differentiation, as shown below.

## Chondrogenic and osteogenic differentiation

Chondrogenesis is a key developmental process in endochondral ossification, which is a fundamental step during skeletal development. Endochondral ossification starts when undifferentiated mesenchymal cells aggregate to form mesenchymal condensation at the point of each future skeletal part by acquiring characteristics of non-hypertrophic chondrocytes. During chondrogenesis, chondrocytes differentiate into proliferating chondrocytes, pre-hypertrophic and then, hypertrophic chondrocytes. Lastly, hypertrophic chondrocytes undergo apoptosis and are replaced by bone. Chondrogenic and osteogenic differentiation of MSCs are complex developmental processes governed by different factors, such as signaling pathways, growth factors and TFs that are required in a precise spatio-temporal sequence to each proper chondrocyte and osteocyte formation.

**Figure 2:**
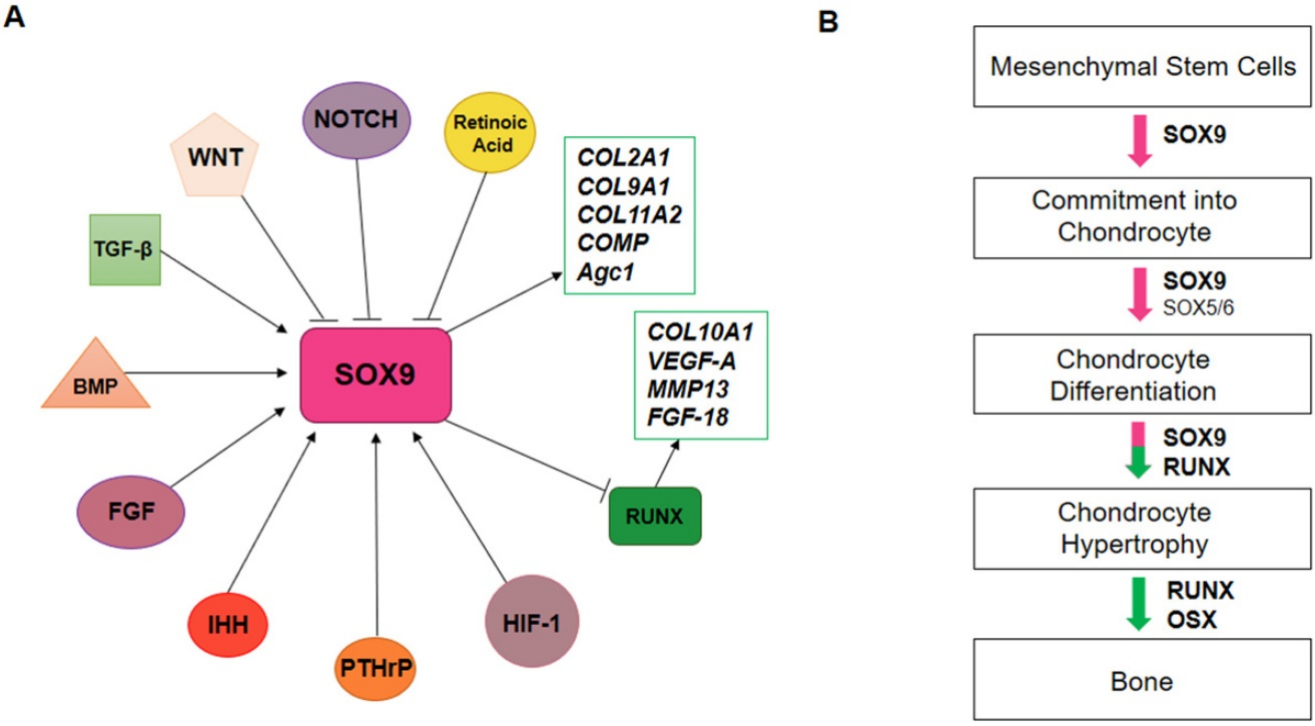
** SOX9 regulation during chondrogenesis. A)** In chondrogenesis, SOX9 is negatively regulated by WNT, NOTCH and retinoic acid pathways. Otherwise, TGF-β, BMP, FGF, IHH, PTHrP, HIF-1 pathways positively regulate SOX9 expression. SOX9 induces *COL2A1, COL9A1, COL11A2, COMP and Agc1* expression and inhibits RUNX expression, which in turn activates COL10a1, MMP13, VEGF-A and FGF-18 genes. **B)** Endochondral ossification is regulated by transcription factors. SOX9 promotes the commitment of mesenchymal stem cells into chondrocytes; SOX5/6/9 promotes chondrocytes differentiation; during chondrocyte hypertrophy, SOX5/6/9 expression decreases and RUNX and OSX expression is activated to promote bone formation.

SRY-Box Transcription Factor 9 (SOX9), the master regulator of chondrogenesis, is highly expressed in mesenchymal osteo/chondro-progenitors, which are proliferating, and pre-hypertrophic chondrocytes, but declines in hypertrophic chondrocytes. SOX9 exerts its effects on chondrogenesis by directing the expression of chondrocyte-specific extracellular matrix genes, such as collagen type II alpha 1 chain (COL2A1), collagen type IX alpha 1 chain (COL9A1), collagen type XI alpha 2 chain (COL11A2), cartilage oligomeric protein (COMP) and aggrecan (Agc1 or ACAN) (Figure 2A). SOX9 regulates chondrocyte gene expression in collaboration with others transcriptional coactivators, SOX5 and SOX6. During chondrocyte hypertrophy, SOX9, SOX5 and SOX6 expression decreases. Furthermore, other transcription factors, such as Runt-related transcription factor 2 (RUNX2) and Runt-related transcription factor 3 (RUNX3), are activated to promote chondrocyte hypertrophy (Figure 2B). RUNX2 directly binds and activates genes like Indian hedgehog (Ihh), COL10A, matrix metalloproteinase 13 (MMP13) and vascular epithelial growth factor (VEGF-A).

Other chondrogenesis related genes are DEC1 and DEC2, two basic helix-loop-helix (bHLH) proteins, which regulate cell differentiation in different tissues including cartilage. Specifically, DEC1 is considered a transcriptional factor cAMP-induced that modulates chondrogenesis 20. DEC2 is a negative regulator of MSC differentiation in chondrogenic lineage, whereas its over-expression downregulates the mRNA expression of chondrogenic markers 21.

TGF-β and bone morphogenic protein (BMP) signaling have a key role in chondrogenic and osteogenic processes. TGF-β/BMP pathways stimulate chondroblasts to proliferate and deposit cartilage-specific extracellular matrix molecules, such as Agc1, COL2A1 and glycosaminoglycans (GAG). Several miRNAs that post-transcriptionally module these signaling pathways by inducing or inhibiting chondrogenesis are reported in Table 1.

**Figure 3:**
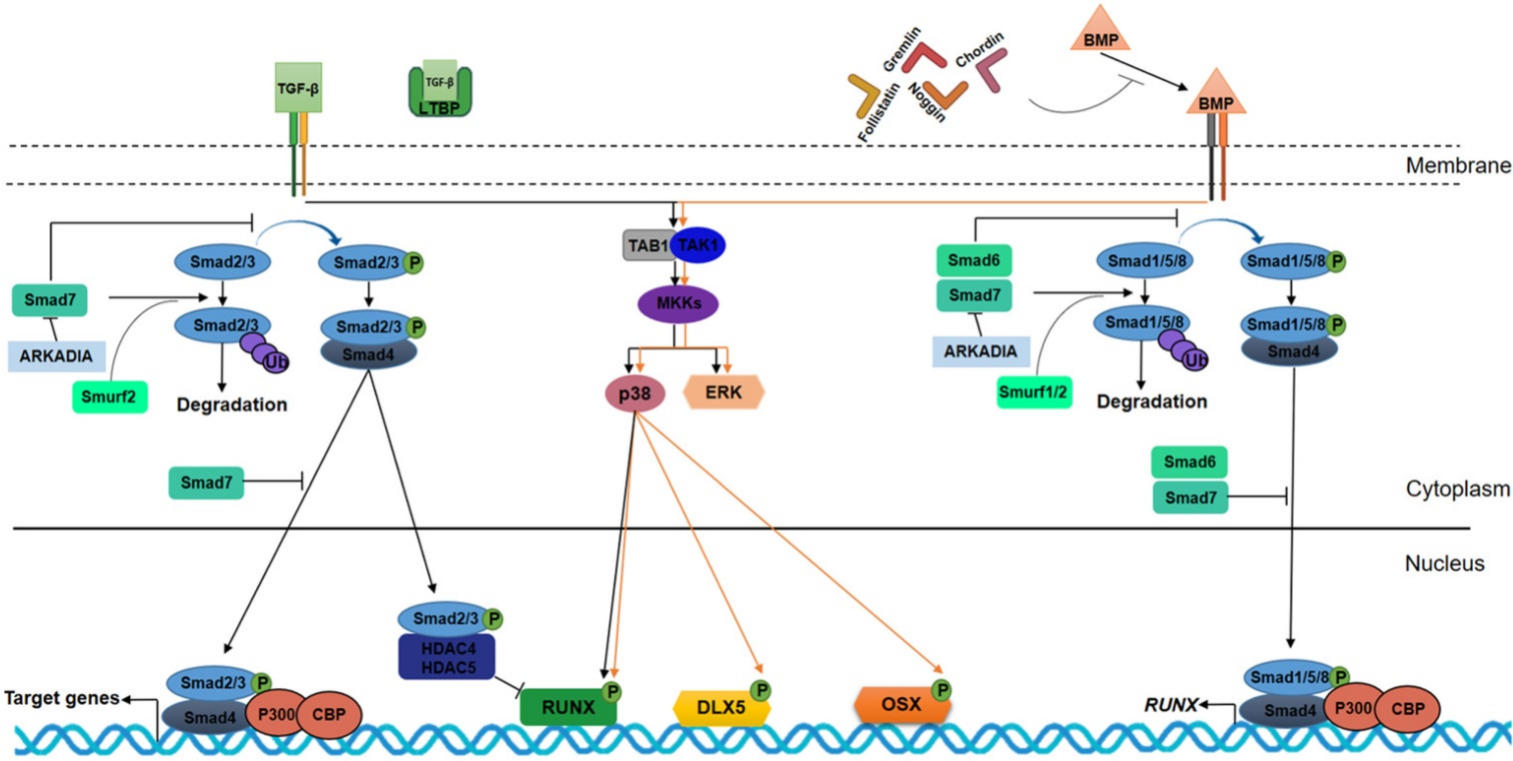
** TGF-β and BMPs signaling pathways in osteogenesis.** TGF-βs and BMPs bind to the extracellular domains of specific receptors and require Sma and Mad related (SMAD) proteins for signal transduction within the cells. TGF-βs and BMPs work through heterodimeric receptors consisting of type I and type II kinase receptors. TβRI/ALK5 is the type I receptor for TGF-βs, and BMPR1A/ALK3, BMPR1B/ALK6 and AcvR1/ALK2 are type I receptors for BMPs. TβRII/TGFBR2 and BMPR2 are the type II kinase receptor for TGF-βs and BMPs, respectively. Upon binding of the ligand to the receptor, the receptor forms homodimeric complexes, resulting in one subunit phosphorylating its partner subunit on the serine/threonine residues. This starts a cascade of events involving SMAD protein phosphorylation. The TGF-β pathway requires SMAD2 and SMAD3, whereas BMP signaling is dependent on SMAD1, 5 and 8. Phosphorylated receptor-regulated SMADs (R-SMAD) react with SMAD4 to create a heterocomplex. This complex, consisting of R-SMAD and common-mediator SMAD (Co-SMAD), enters the nucleus and controls transcription by binding to target gene promoters. Phosphorylated R-SMAD (R-SMAD-Pi) interacts with SMAD4 protein, then translocates into the cell nucleus where AMP response element binding protein (CREB)-binding protein (CBP) and P300 coactivators are recruited which regulate gene targets. SMAD2/3-Pi in the nucleus and SMAD4-unlinked recruit HDAC4 and HDAC5 by inhibiting RUNX2 and OSX expression. In the Non-Smad-dependent signaling pathway, TGF-β activates kinase 1 (TAK1) and TAK1-binding protein 1 (TAB1) to initiate the MKKs (MAPK pathway member-encoding genes kinases)-p38 MAPK or -Erk (extracellular signal regulated kinase) signaling cascades. Interaction between BMPs and their receptors result in comparable events triggered by TGF‐β. In BMP signaling, R-SMAD (SMAD1/5/8) bind SMAD4 to translocate into the nucleus, where they induce RUNX2 expression. Similarly, Non-Smad-dependent BMP-stimulated pathway induces DLX5, RUNX2, and OSX expression and leads to osteoblast and osteocyte differentiation. Latent TGF-beta binding proteins (LTBPs) bind TGF-β and prevent its interaction with TβRI and TβRII receptors; otherwise, Noggin, Chordin, Gremlin and Follistatin bind to BMP proteins and block their signaling cascades. In TGF- β signaling, SMAD7 inhibits SMAD2/3 translocation into the nucleus, and, alongside Smurf2 induces R-SMAD proteasome-mediated degradation. Similarly, in BMP signaling, SMAD6/7 inhibit SMAD1/5/8 translocation into the nucleus and, alongside Smurf1/2 induces SMAD1/5/8 and RUNX2 proteasome-mediated degradation. In turn, SMAD6 expression is regulated by RUNX2 in a negative feedback loop. ARKADIA protein positively regulates osteoblast differentiation by binding SMAD6/7 proteins.

In osteogenesis, the TGF-β pathway is activated in the early stages, allowing transition from osteo-progenitor cells to immature osteoblasts to take place through osteogenic marker expression (Figure 3). In osteogenic differentiation, the homeodomain protein Distal-less Homeobox 5 (DLX5) is an activator of master TFs in osteogenesis: RUNX2 and the zinc finger transcription factor OSX. OSX, also known as Sp7, is an osteoblast-specific transcription factor that induces osteoblast differentiation and bone formation. OSX is a downstream RUNX2 gene, which is considered to be a fundamental osteogenic TF that regulates osteoblastic genes, such as osteopontin (OPN), osteocalcin (OCN), osteonectin (ON) and collagen type 1 (COL1). Some evidences have demonstrated that OSX is also regulated via RUNX2-independent mechanism.

Indeed, the homeobox gene MSX2, which is up-regulated by BMP2, induces OSX over-expression, thus favoring osteoblastic differentiation 22.

In addition to TGF‐β/BMP signaling cascades, Wingless/Int-1 (Wnt)/β-catenin signaling pathway is crucial for MSC osteogenic differentiation by guiding MSCs into pre-osteoblast cells. Twist Family BHLH Transcription Factor 1 (TWIST-1) gene is a target for the Wnt/β-catenin cascade, which is required for cranial bone lineage commitment. Interestingly, TWIST-1 exerts its function by maintaining Wnt responsiveness. TWIST-1 knockdown leads to cranial bone agenesis, as well as eye and palate malformation 23. TGF-β/BMP and Wnt/β-catenin signaling pathways affect each other in regulating skeletal tissue formation *in vivo* and *in vitro*. Furthermore, several miRNAs, which are reported in Table 2, post-transcriptionally module these signaling pathways by inducing or inhibiting osteogenesis.

## MiRNAs in chondrogenic differentiation

### MiRNAs positively regulating chondrogenesis

Among miRNAs regulating chondrogenesis, miR-140 is the most-highly studied. MiR-140 targets *Sp1* in order to maintain chondrocyte proliferation 24. Histone deacetylase 4 (*HDAC4*) and ADAM Metallopeptidase with Thrombospondin Type 1 Motif 5 (*ADAMTS‐5*) have also been proposed as target genes for miR-140 24. MiR-140-3p and miR-140-5p are up-regulated in human MSCs (hMSCs) during chondrogenesis and target genes identified in terminal hypertrophic differentiation. Specifically, miR-140-5p targets the 3'UTC of Ras-related protein Ral-A (*RALA*), enhancing SOX9 and ultimately, *ACAN* 25. MiR-520d-5p also promotes hMSC chondrogenesis and regulates chondrocyte metabolism by targeting Histone Deacetylase 1 (*HDAC1*) 26. MiR-574-3p has been found to be early up-regulated during chondrocyte differentiation. The retinoid X receptor (*RXRα*) has been identified as a direct target for miR-574-3p in hMSCs, whereas it seems that this miRNA is also involved in a negative feedback loop required for chondrocyte lineage maintenance and MSC multipotency 27. In rat bone marrow stromal cells (BM-MSCs), the expression of early chondrogenesis markers SOX9, COL2A1 and ACAN was stimulated after miR-127-5p transfection, while it decreased in late markers COL10A1 and RUNX2. Therefore, miR-127-5p upregulation seems to promote chondrogenesis, thus preventing hypertrophic differentiation 28.

### MiRNAs negatively regulating chondrogenesis

MiR-124 directly targets and inhibits the Nuclear Factor of Activated T Cells 1 (*NFATC1*). The latter directly interacts with the *SOX9* promoter 29. During chondrogenesis, miR-124 is downregulated by promoter methylation. MiR-29a was previously identified as a chondrogenesis suppressor in MSCs 29. MiR-29a targets the 3'UTR of transcription factor Forkhead Box Protein O3 (FOXO3A), which is up-regulated during chondrocyte differentiation. FOXO3A regulates SOX9, ACAN and COL2A1 expression during chondrogenesis 25. MiR-30a regulates chondrogenic differentiation in hMSCs 30, while miR-30b regulates chondrogenic differentiation in mouse embryo-derived stem cells C3H10T1/2 31.

In addition, decreased miR-221 induced an increase in chondrogenic markers, such as COL2A1, ACAN and SOX9 by targeting *FOXO3*. MiR-221 silencing promotes *in vitro* MSCs differentiation towards chondrocytes in the absence of TGFβ, enhancing *in vivo* cartilage repair. Antago-miR-221 treatment has been observed to restore FOXO3 in a context of tissue degeneration and inflammation 32. MiR-182-5p is an inhibitor of chondrogenesis; it targets Parathyroid Hormone like Hormone (*PTHLH*) and its knockdown has been correlated to an increase in chondrogenic markers, such as SOX-9 and COL2A1 33. Another miRNAs inhibit chondrogenesis by directing targeting *SOX9* is miR-101. SOX9 expression was negatively regulated by miR-101 in rat chondrocytes; it promoted chondrocyte late differentiation in rat BM-MSCs 34.

MiR-1247 targets cartilage transcription factor SOX9 and, in a negative feedback loop, SOX9 inhibits miR-1247 expression in human chondrocytes 35. Upon miR-1247 over-expression, *COL2A1* and miR-140 levels are significantly reduced. MiR-145 has also been reported to be downregulated during murine and human MSC chondrogenic differentiation. It directly targets the 3'UTR *SOX9* 36. Some miRNAs, such as miR-495 and miR-194, inhibit chondrocyte MSCs differentiation by targeting *SOX9* or *SOX5* 37.

### TGF-beta signaling pathway regulation in chondrogenesis

TGFβ1, 2 and 3 are known as typical MSC chondrogenesis inducers. MicroRNA-337 is associated with chondrogenesis by regulating TGF-β type II receptor (TGFBR2) expression. MiR-337 negatively targets *TGFBR2*, which has an important role in cartilage development. Indeed, during the maturation phases of endochondral ossification the expression of miR-337 is substantially downregulated 38. MiR-193b regulates early chondrogenesis by targeting *TGFBR2* 39, while loss of miR-17-92 seems to reduce mesenchymal progenitor cell (MPC) proliferation by depressing TGF-β signaling 29.

### BMP signaling cascade regulation in chondrogenesis

BMPs are members of the TGF-β family. MiR-199a adversely regulates early chondrocyte differentiation by directly targeting *SMAD1* in the BMP signaling pathway. MiR-155 targets the 3′ UTR of multiple components in BMPs, including SMAD1 in epithelial cell line A549 40. MiR-92a has also been identified in the regulation of the BMP signaling pathway. Noggin3 (*Nog3*), a BMP antagonist, was identified as a direct target of miR-92a 38.

### RUNX2 regulation in chondrogenesis

MiR-30 family members (miR-30a, -30b, -30c, and -30d) are able to target both *RUNX2* and *SMAD1*, therefore preventing osteogenesis in mature chondrocytes 40. MiR-455-3p also targets *RUNX2* in ATDC5 cell lines and promotes early chondrocyte differentiation and inhibits chondrocyte maturation. It has been suggested that miR-455-3p increases *COL2A1* promoter acetylation thereby increasing gene transcription and promoting chondrocyte differentiation 29. MiR-320 targets *RUNX2*. Its expression was found to be elevated in ATDC5 cells, whereas it seems to be implicated in reducing cartilage hypertrophy and degeneration. MiR-320 also targets *MMP13* and reduces its levels 29. During growth plate development, miR-1 plays an important role in the regulation of chondrocyte differentiation by targeting HDAC4, which in turn regulates chondrocyte hypertrophy by inhibiting *RUNX2* 41.

### MMP-13 regulation in chondrogenesis

MMP-13 has been shown to be regulated by miRNAs including miR-9, miR-27b and miR-381. MiR-27b binds directly to the 3′ UTR of *MMP-*13 in nucleus pulposus (NP) cells 42*.* MiR-381 has been identified as a regulator of chondrogenesis related genes. It was found to be up-regulated in late stages during *in vitro* chondrocyte differentiation. MiR-381 increases *MMP-13* and *RUNX2* expression and decreases *COL2A1* expression in ATDC5 cells. It has been shown to target HDAC4 in SW1353 cells 29,43. MiR-9 negatively regulates MMP-13 and protogenin (*PRTG*) expression. MiR-9 abrogation results in increased PRTG levels, inhibited cell proliferation and survival in chondrogenic progenitors and articular chondrocytes 41.

### VEGF regulation in chondrogenesis

Some miRNAs positively regulating chondrogenesis through VEGF are miR-424 which is able to repress *VEGF*, vascular endothelial growth factor receptor 2 (*VEGFR2*) and fibroblast growth factor receptor 1 (*FGFR1*) in early chondrogenesis 44. MiR-15 and -16 regulate VEGF expression and were downregulated during hypoxia 45. MiR-107 targets phosphatase and tensin homolog (*PTEN*), modulating chondrocyte proliferation, apoptosis and extracellular matrix synthesis. Upregulation of miR-107 inhibits angiogenesis and VEGF expression and promotes ACAN and COL2A1 expression, attenuating MMP-13 and MMP-9 expression in C28/I2 cells 46. MiR-210 promoted COL2A1 production from meniscus cells through *VEGF* and *FGF2* upregulation in synovial cells by targeting different genes including Homeobox A 1/9 (HOXA-1/9) 41. Finally, miR‐320 negatively regulates VEGF expression through ERK 1/2 and regulates both ADAMTS5 and HOXA10, which in turn regulate MMP13 through RUNX2 47. Studies involving hMSCs into chondrocytes found let-7b as a putative miRNA regulator of *VEGF* 48.

Another miRNA which negatively regulates chondrogenesis was miR-29a which targeted *VEGF* mRNA through binding to its 3′-UTR 49. VEGF and anti-angiogenic miR-20a, miR-106a-5p, and miR-20b were also studied. MiR-20a is a critical negative regulator of chondrogenic differentiation inhibiting autophagy by targeting autophagy Related 7 (*Atg7*) 50. Both, miR-17-5p and miR-20a promote chicken cell proliferation by targeting Mitogen-Activated Protein Kinase Kinase Kinase 2 (*MAP3K2*) and in consequence upregulating c-Myc. MiR-20b targets the early chondrogenic-related gene Endothelial PAS Domain Protein 1 (*EPAS1*), which is involved in embryonic organ development, by negatively regulating chondrogenic differentiation 44. The miR-17-92 cluster and its paralogs, including miR-106a-92 and miR-106b-25, are highly expressed in myeloid progenitors and target Sequestosome 1 (*SQSTM1/p62*) 50. Clusters miR-106b-25 and miR-17-92 were involved in TGF-β signaling modulation 41. MiR-193b downregulates factors such as COL2A1, ACAN and SOX9, while it targets Dimethylarginine Dimethylaminohydrolase 1 (*DDAH1*), which in turn results in decreased VEGF secretion *in vivo* 41.

## MiRNAs in osteogenic differentiation

### RUNX2 regulation in osteogenesis

A panel of 11 miRNAs that target *RUNX2* expressed in a lineage-related pattern in MSCs types, i.e., miR-23a, miR-30c, miR-34c, miR-133a, miR-135a, miR-137, miR-204, miR-205, miR-217, miR-218 and miR-338 has been reported. All these *RUNX2*‐targeting miRNAs (except miR‐218) significantly inhibit osteoblast differentiation 51. In addition, other miRNAs that are related to the *RUNX2* gene during MSCs differentiation to pre-osteoblasts, such as miR-23b, miR-30b, miR-143, miR-203 and miR-221, have also been identified 52. For instance, a recent study has shown that miR-30 down-regulated *RUNX2* by interacting with the 3′-UTR of *RUNX2* in adipose-derived MSCs (hASCs) 53. Li et al., reported that miR-133 inhibit *RUNX2*-mediated osteogenesis 54. The over-expression of miR-23a in mouse osteoblast-like cells (MC3T3-E1) inhibits osteogenic differentiation by inhibiting *RUNX2* 51. In contrast, miR-23a cluster has been indicated as promoting osteogenic differentiation by modulating TGF-β signaling pathway by targeting PR domain-containing 16 (*Prdm16*) in MC3T3‐E1 cells 55. MiR-133a is reported as being an important regulator in osteogenesis, as its expression is down-regulated in BMP-induced osteogenesis and can target and suppress *RUNX2* expression in order to inhibit osteoblast differentiation 54. A recent study has demonstrated inhibition of osteoblast differentiation marker expression, ECM mineralization and ALP activity, by miR-133a-5p in MC3T3-E1 cells 56. MiR-135a strongly inhibits RUNX2 protein expression in both MC3T3-E1 and ATDC5 cells 51. A recent study has reported that miR‑135a‑5p expression was significantly decreased during osteogenic differentiation *in vitro* in mouse myoblast cell line (C2C12), whereas it was significantly increased in post-menopausal females with osteoporosis 57. Recently, Kong et al., have reported that miR-137-3p directly targets *RUNX2* and its silencing promotes both osteogenesis and angiogenesis *in vitro* and *in vivo* 58.

In a recent study, it has been reported that miR‐203 can induce the upregulation of RUNX2 and OCN. The authors have suggested that miR‐203, which is up-regulated during osteogenic differentiation, could promote proliferation, migration and osteogenic differentiation of MC3T3‐E1 cells. Furthermore, it has been proposed that miR‐203 might affect MC3T3‐E1 cells by upregulating MSX2, along with MAPK/STAT pathway activation 59. An *in vitro* study has demonstrated that miR-410-3p suppresses osteogenic-like differentiation by directly targeting MSX2 in human renal interstitial fibroblasts (hRIFs) 60.

Huang et al., reported that two homologous miRNAs, miR-204 and miR-211, suppress *RUNX2* expression in MPCs and BM-MSCs promoting adipogenesis 61. Hu et al., reported that miR-205 expression was down-regulated in a time-dependent manner during BM-MSC osteo-induction. Furthermore, these authors found that mir-205 could regulate protein expression of special AT-rich sequence-binding protein 2 (*SATB2*) and *RUNX2* 62. An *in vitro* analysis revealed that the over-expression of miR-338-3p can inhibit the expression of osteoblast differentiation markers such as OSX, thus reducing osteoblast differentiation. Bioinformatic analysis and dual luciferase assays confirmed that miR-338-3p can repress gene expression by targeting *RUNX2* and *FGFR2* 63. It has also been reported that miR‐218 decreases *RUNX2* expression in undifferentiated human-derived dental stem cells (hDSCs) 64. Suppression of miR-222-3p activity has been reported as promoting osteogenic differentiation of human BM-MSCs by regulating the *SMAD5-RUNX2* signaling axis 65. It has been demonstrated that miR-375 expression levels decrease in a C2C12 cell model of osteogenic differentiation. Over-expression of miR-375 inhibited the activity of pivotal osteoblast markers, such as ALP, OCN and collagen, type I, α 1 (COL1A1) 66. However, it has been demonstrated that miR‐375 over-expression enhances hASCs osteogenesis both *in vitro* and *in vivo* by directly targeting *DEPTOR* (mTOR inhibitor) and Yes‐associated protein1 (*YAP1*) in these cells 67.The circadian rhythm helps coordinate bone formation and resorption in the skeleton. MiR-433 was previously shown to target *RUNX2* in C3H10T1/2 cells 68 and seems to help maintain the circadian rhythm in osteoblasts by regulating sensitivity to glucocorticoid receptor signaling 69. MiR-505 expression was down-regulated during osteogenic differentiation in MC3T3-E1 cells 70. Recently, a study has indicated that miR-628-3p was up-regulated in patients with atrophic non-union and exerted an inhibitory effect on osteogenesis through the suppression of its target gene, *RUNX2. In vitro*, miR-628-3p mimics attenuated the osteogenic differentiation of MG63 cells 71. Interestingly, it has been reported that nicotine inhibits periodontal ligament-derived stem cells (PDLSCs) proliferation, migration, and osteogenic differentiation by increasing miR‐1305 levels. Restoration of miR-1305 relieves the inhibitory effect of nicotine on PDLSCs proliferation, migration and osteogenic differentiation by upregulating RUNX2 levels 72.

Under hypoxia, TWIST-1 is induced and acts as a transcription repressor of RUNX2 by binding to the E-box located on the RUNX2 promoter, thus, suppressing osteogenesis in hMSCs 73. In addition to regulating protein-coding genes, it has recently become evident that TWIST-1 can also regulate miRNAs 74. MiR-376c-3p targets IGF1R inhibiting BMSC osteogenic differentiation whereas it has been identified as a TWIST-1-regulated miRNA, which plays an important role in the osteogenesis of bone precursor cells and can mediate TWIST-1 inhibition of osteogenesis. Furthermore, overexpression of miR-376c-3p in TWIST-1 haploinsufficient calvarial cells can decrease the aberrant osteogenesis of these cells, which contributes to increased calvarial bone volume and premature fusion of coronal sutures 75.

### Sp7 regulation in osteogenesis

Expression of the Sp7, a zinc finger transcription factor and critical regulator of osteoblast mineralization, was found to be inversely correlated to miR-93 in primary mouse osteoblasts from the calvaria of C57BL/6 mice 76. By stimulating and/or inhibiting miR-93-5p expression in human BM-MSCs, Zhang et al., confirmed that osteogenic differentiation-related indictors, including *OSX*, were decreased by miR-93-5p 77. MiR-145, a Sp7 regulator, suppresses the osteogenic differentiation of C2C12 and MC3T3-E1 cells 78. It has been reported that miR‐145 and miR‐34c cooperatively inhibit osteoblast differentiation in MC3T3‐E1 cells 79. Liu et al., 80 reported that miR‐143 and miR‐145 regulate odontoblast differentiation by binding the 3′‐UTR of *Sp7*. It has been reported that miR-132 expression also decreases during osteogenic differentiation of umbilical cord mesenchymal stem cells (UC-MSCs). MiR-132 upregulation inhibited the osteogenic differentiation of UC-MSCs by attenuating the activity of the Wnt/β-catenin signaling pathway targeting *β-catenin* and downregulating OSX levels 81. MiR-214 expression decreases gradually during osteoblast differentiation and plays an important role in different stages of bone formation and resorption by targeting *Sp7,* Activating Transcription Factor 4 (*ATF4*)*, β-catenin and FGFR1* 82*.* A study reported that moderate exercise could inhibit miR-214 expression in bone 83. MiR-637 expression in hMSCs is indispensable for maintaining the balance of adipocytes and osteoblasts 84.

### BMP regulation in osteogenesis

Bone morphogenetic proteins BMP and BMPRs are known as important factors for skeletal development, regeneration and homeostasis 85.

Several miRNAs negatively regulate BMP-induced osteoblastogenesis. For instance, mir-93-5p, mir-98 and miR-140-5p inhibit osteogenic differentiation in human MSCs by direct targeting *BMP-2* 77,86,87. In human periodontal ligament fibroblasts (hPDLFs) miR-140 is able to inhibit osteodifferentiation. Indeed, the authors have suggested that miR-140 suppresses osteogenic differentiation of hPDLFs by targeting *RhoA,* an important member of the small GTPase of RHO family that participates in key processes of osteogenic differentiation 88.

In PDLSCs, miR-106a-5p targets BMP-2 and E2F Transcription Factor 5 (E2F5) inhibiting the osteogenic differentiation 89. It has been demonstrated that miR-370 attenuates the osteogenic differentiation of BMP2-stimulated MC3T3-E1 cells by targeting *BMP-2* and erythroblastosis virus E26 oncogene homolog 1 (*Ets1*) 90. Recently, a study was carried out using bioinformatics tools which revealed *BMP-2* to be a potential target gene for miR-370-3p in hASCs 91. It has also been reported that mir-195-5p inhibits osteogenesis in human PDLSCs by targeting BMP receptor type I (*BMPR1A*) 92. Furthermore, it has been shown that miR-100 and mir-153 attenuate osteogenic differentiation of hMSCs by targeting *BMPR2* 93,94.

### SMAD regulation in osteogenesis

MiR-144-3p inhibits the proliferation of C3H10T1/2 cells by arresting cells at the G0/G1 phase. Results from bioinformatics analysis, luciferase assays and western blotting have demonstrated that miR-144-3p directly targets *SMAD4* 95. It has been reported that miR-146a negatively regulates the osteogenesis and bone regeneration from hASCs both *in vitro* and *in vivo* 96. A single study reported that miR-203-3p targets the 3′-UTR of *SMAD1* mRNA; interestingly, miR-203-3p was identified as being up-regulated in the diabetic group and when compared to normal rats 97.

MiR-26a is required for skeletal muscle differentiation/regeneration in mice and it is up-regulated during osteogenic differentiation in unrestricted somatic stem cells (USSC) and shares target genes which inhibit osteogenesis with miR-29b. These miRNAs accelerate osteogenic differentiation and are likely to be mediated by osteo-inhibitory proteins such as CDK6 and HDAC4 98.

Interestingly, Su et al., identified miR-26a as a positive regulator in BM-MSC osteogenic differentiation, but as a negative regulator in hASCs osteogenic differentiation 99. It has been reported that the expression levels of some miR-30 family members, such as miR-30a, -30b, -30c, and -30d, are significantly down-regulated during osteoblast differentiation; for instance, miR-30 mediates osteogenesis inhibition by targeting *SMAD1* and *RUNX2* in MC3T3-E1 40. In early osteogenesis, BMP-2 signaling appears to downregulate miR-133 and miR-135 which suppress two essential TFs for osteogenesis, *RUNX2* and *SMAD5*, forming a transcriptional complex 100.

Finally, a recent study has reported that miR-497-5p increases in human stem cells from apical papilla (SCAP), osteo/odontogenic differentiation. Furthermore, bioinformatic analysis and dual luciferase reporter assay have identified SMAD specific E3 ubiquitin protein ligase 2 (*Smurf2)* as a negative osteogenesis. This gene is a target of miR-497-5p, demonstrating that miR-497-5p acts as a positive regulator of osteo/odontogenic differentiation in SCAP 101.

### SMAD7 regulation in osteogenesis

The literature shows that *SMAD7* is one of the target genes for miR-21a and *SMAD7* has an antagonistic role in TGF-β1 signaling 102. Indeed, miR-21 promotes BM-MSCs bone formation and this process is regulated, in part, by the SMAD7-SMAD1/5/8-RUNX2 pathway 102. Sanjeev et al., indicated that treatment with phytol, an acyclic unsaturated diterpene alcohol and a secondary metabolite derived from aromatic plants, promotes osteoblast differentiation in C3H10T1/2 cells via *RUNX2* due to down-regulation of *SMAD7* by miR-21a 103. In addition, pulsed electromagnetic fields (PEMFs) could mediate its effects on bone metabolism by activating the TGF-β signaling pathway and stimulating miR-21-5p expression in human BM-MSCs 104. It has been reported that miR-17-5p, which is located in the miR-17/92 cluster and has important roles in various cancers, regulated osteoblastic differentiation and cell proliferation by directly targeting *SMAD7* in human BM-MSCs 105.

MiR-590-5p has been reported to stimulate osteoblast differentiation of C3H10T1/2 and MG63 cells by indirectly protecting and stabilizing the RUNX2 protein by targeting *SMAD7* 106.

### HDAC4 regulation in osteogenesis

Over-expression of miR-29a and miR-29b can block *HDAC4* expression and enhance osteoblast differentiation in primary BM-MSCs and MC3TC-E1 cells 107,108. Delivery of osteogenesis-promoting miRNAs is a promising approach for enhancing bone regeneration 109. For this reason, Liu et al., have developed an R9-LK15/miR-29b nano-complex that has high transfection efficiency and promotes osteogenic differentiation 109.

### Wnt/β-catenin signaling pathway

Wnt family is formed of 19 secreted glycoproteins that are involved in various biological processes, such as regulation of MSCs fate and includes two distinct canonical (β-catenin dependent) and non-canonical branches. The effects of Wnt signaling pathway on osteoblastogenesis has been shown via overexpression and knockdown of Wnt pathway ligands and components 110.

Many members of the sirtuin family have been confirmed as being related to Wnt signaling. Recently, it has been indicated that the sirtuin family is closely related to osteogenic differentiation 111. In hASCs, the over-expression of miR-130a-3p induces a significant increase in RUNX2 mRNA targeting SIRT7 112.

Wnt6 and Wnt10, which act as MSCs fate regulators, were identified as targets of miR-378. The use of miR-378-mimics could suppress osteogenesis in hMSCs, whereas anti-miR-378 allows for osteogenic differentiation. Thus, miR-378 inhibits osteogenesis and bone formation by inactivating Wnt/β- catenin signaling 113.

## MiRNAs in bone fractures and bone diseases

Fracture healing is a proliferative physiological process whereby the body facilitates bone fracture repairs 114. In the first phase of this process, MSCs are recruited to the fracture site and differentiated into fibrocytes, chondrocytes or osteoblasts. Subsequently, in the following phase, ECM and hard callus are generated, alongside angiogenesis and revascularization. At last, during the late phase, the callus is continuously remodeled to respond to the biomechanical and biological demands of the new bone 115. MiRNAs play a critical role in many physiological processes, as reported above. Moreover, they have recently emerged as key regulators in the complex process of fracture healing. Li et al. 116 have highlighted high miR-214-5p expression in the plasma of patients with hand or intra-articular calcaneal fractures and demonstrated the importance of miR-214-5p down-regulation which resulted in the enhancement of osteoblastic cell viability and resistance to apoptosis 116. *SMAD6* is known to be a critical feedback inhibitory governor of BMP/SMAD signaling. In silico prediction has indicated that miR-186 is a regulatory miRNA of *SMAD6* as far as fracture healing is concerned 117. In addition, miR-186 could activate the BMP signaling pathway to promote fracture healing by inhibiting *SMAD6* in a mouse model of femoral fracture 117. Lee and colleagues have shown the meaningful therapeutic effect of miR-29b-3p in femoral fracture healing; this *in vivo* study showed that a single injection of miR-29b-3p, delivered via a microbubble-ultrasound system, 14 days post fracture improved healing outcome 118. The presence of 134 miRNA was detected in plasma from 4 patients with trochanteric fractures compared with 4 healthy controls 119. Moreover, the levels of six miRNAs (miR-16, miR-19b-1, miR-25, miR-92a, miR-101, and miR-129-5p) were also shown to be dysregulated. Shi et al., 120 investigated whether miR-218 can have an impact to bone regeneration. Specifically, when injected at the fracture site of mouse femoral transverse fractures, miR‐218 overexpressing cells significantly increased bone volume at 2 and 4 weeks post fracture compared to the control group 120.

In recent years, there has been an increase in the prevalence of bone diseases due to population aging. Bone diseases affect millions of people worldwide. However, these pathologies are more common among the elderly 121. Age-related bone and osteoarticular diseases include different pathologies such as metabolic bone diseases, osteoporosis (OP) and osteoarthritis 121. Among these, OP, a chronic skeletal disorder characterised by increasing of bone fragility and susceptibility to fracture, is the most common bone disease in humans and represents one of the greatest public health problems 122. OP affects about 200 million people all over the world; it affects both sexes and all races. Fractures are the most serious consequence associated with OP, causing significant burdens in terms of health care costs, morbidity and mortality annually 121. Currently, the gold standard for diagnosing OP is bone mineral density (BMD) analysis which is measured by means of dual X-ray absorptiometry (DXA). BMD measurements of the hip and spine can be used to determine/confirm the diagnosis of OP and predict future fracture risks and monitor patients 122. Diagnostic approaches to this pathology have limited sensitivity and specificity, also as far as monitoring disease progression or treatment is concerned. Therefore, new biomarkers are needed to improve the management of OP patients.

Despite the critical importance of miRNAs in regulating various cell functions, their role in OP has not yet been investigated in depth in well-characterized human bone. However, identifying specific miRNAs associated with OP could provide additional information, mainly by integrating multiple signals, which, when amalgamated imaging and biochemical markers, could improve a standard diagnostic pathway and increase prognostic potential 121.

In recent years, miRNAs have been widely studied as biomarkers in clinical practice thanks to their key modulatory role in multiple biological functions and their association with numerous physiological and pathological conditions 123. As reported above, altered expression levels of miRNAs were found to be associated with bone pathologies and tumours in several studies. Although miRNAs have been identified as potential biomarkers, their evaluation for clinical application requires the standardization of all aspects of miRNA analysis from discovery to validation. Standardization of pre-analytical, analytical, and post-analytical protocols would allow all the variables that can introduce bias in the detection of biomarkers to be monitored 124.

However, there are some drawbacks for miRNAs as biomarkers, such as lack of standardized protocols for the management of all the subject-related factors (age, gender, lifestyle, diseases) and sample-associated variables (matrix/source, sample collection and handling), that may affect the concentration/expression level of the miRNA and its measurement, respectively 125. An important source of variability in the analytical phase of miRNA evaluation is the quantification platform used (PCR, next generation sequencing and microarray). Results obtained using different technologies show significant differences in evaluating miRNA levels. Therefore, it is fundamental for analytical protocols and the platform used to be standardized 123. When considering the post-analytical phase, the most challenging problem is represented by the absence of a standardized methodology for the reference gene selection alongside the normalization strategy used for miRNA quantification 124.

Though there are still several problems yet to be solved in the miRNA validation process, the use of miRNAs as biomarkers in clinical application and research has shown different advantages. Specifically, miRNAs can be isolated and evaluated in human biofluids (e.g., urine, plasma and serum) with non-invasive procedures 123. Moreover, miRNA detection and quantification are based on PCR methods that are characterized by reproducible results with elevated specificity and sensitivity. Lastly, miRNAs in human biofluids, can be found to be both bind to proteins or enclose in exosomes, ectosomes or in lipoproteins, thus showing elevated stability.

Interestingly, TAmiRNA, a European firm, leader in miRNA-based diagnostics, has recently commercialized a product named Osteo-miR panel, using a set of 19 miRNAs, that is able to identify the risk of first fracture in post-menopausal osteoporosis and type-2 diabetes in women 126.

### MiRNAs as circulating biomarkers in OP and osteoporotic fractures

Several studies have reported that circulating miRNAs, which have been analysed and validated in serum or plasma as well as in whole blood, could be used as prognostic or diagnostic markers for discriminating OP patients from non-OP subjects.

Circulating levels of miR-133a and miR-21 were analysed in 120 plasma samples from Chinese postmenopausal women. Their level resulted, respectively, as increased and decreased in plasma from OP and osteopenia patients compared to healthy subjects and both are associated with BMD 127. Li et al, analysed miR-133a expression levels in serum samples from ten postmenopausal Chinese women affected by OP compared to the healthy control group. In this study miR-133a was significantly up-regulated in postmenopausal osteoporotic women compared to the control group, and negatively correlated with lumbar spine BMD, according to previous studies 127,128. In addition, this study highlights that miRNA-133a is involved in regulating postmenopausal OP by promoting osteoclast differentiation 128.

MiR-21 down-regulation has been identified in BM-MSCs isolated from women with postmenopausal OP 127. Nine miRNAs, namely miR-21, miR-23a, miR-24, miR-93, miR-100, miR-122a, miR-124a, miR-125b and miR-148a were shown to be significantly increased in serum from 30 patients with OP, when compared to 30 non-osteoporotic controls 129. In the same study, five (miR-21, miR-23a, miR-24, miR-25, miR-100 and miR-125b) of these nine validated miRNAs also resulted as up-regulated in the bone tissue of osteoporotic patients 129. An additional paper validated the increased serum levels of miR-148a, miR-125b, miR-124a, miR-122a, miR-100, miR-93, miR-24, miR-23a and miR-21 in another independent cohort of fractured postmenopausal OP women 130. MiR-30b-5p, miR-103-3p, miR-142-3p and miR-328-3p were found to be down-regulated in postmenopausal OP after animal experiments and serum validation analysis of miRNA from OP patients. All of the aforementioned showed positive correlations with BMD 131. Five miRNAs, including of miR-590-5p, miR-194-5p, miR-151a-3p, miR-151b and miR-130b-3p were increased in whole blood samples collected from postmenopausal OP Chinese women when compared to the osteopenia group 132. Out of these, miR-194-5p circulating levels were strongly increased and negatively correlated with BMD. In addition, miR-194-5p was functionally associated with eight OP-related pathways suggesting that this miRNA may affect TGF-β and Wnt signalling pathways, which were shown to play critical roles in the pathology of postmenopausal OP 132.

The analysis of miRNAs isolated from 74 serum samples derived from postmenopausal women, divided into OP and control groups, showed that miR-148a-3p expression levels were significantly increased in the OP patient group compared to the controls 133. MiR-148a could have different roles in OP pathogenesis. Specifically, it promotes the differentiation of osteoclast targeting the gene V-mafmusculo aponeurotic fibrosarcoma oncogene homolog B (*MAFB*), which is a transcription factor negatively regulating RANKL-induced differentiation of osteoclasts 133.

Other potential circulating markers for postmenopausal OP identified in serum are miR-23b-3p and miR-140-3p, which are increased in OP patients compared to control groups and are associated with low bone mineral density 134. Interestingly, target genes of both miRNAs have been reported as being related to osteoblast differentiation *in vivo* or *in vitro* studies 134. Similarly, in another study increased levels of serum miR-483-5p were found in OP women vs. non-OP controls 135. Additionally, Insulin-like growth factor-2 (*IGF2*) was found to be directly targeted by miRNA-483-5p, suggesting that this miRNA is involved in the pathogenesis of OP by promoting osteoclast differentiation 135.

A recent study, conducted on a cohort of postmenopausal OP women and their control group, has reported increased levels of miR-338 cluster, including miR-3065-5p and miR-338-3p. *In vitro* and *in vivo* studies have suggested that *RUNX2* and *SOX4* could be two critical functional targets of the miR-338 cluster by mediating its regulation during bone formation 136. Lastly, abnormal miR-135b-5p expression in the bone tissue of patients with OP has suggested that this miRNA could be involved in OP occurrence and development by inhibiting osteogenic differentiation and osteoblast proliferation by targeting *RUNX2* 114.

Vertebral fractures characterize OP. Postmenopausal women with and without vertebral fractures were included in a single-site, case-control, observational, cross-sectional study at a University hospital 137. The study identified seven significantly up-regulated miRNAs (miR-375, miR-532-3p, miR-19b-3p, miR-152-3p, miR-23a-3p, miR-335-5p and miR-21-5p) in patients with vertebral fractures and low BMD compared to low BMD and healthy individuals, regardless of OP treatment.

## MiRNAs and bone tumours

MiRNAs have been drawing attention as novel biomarkers for the diagnosis, prognosis and treatment of bone tumours, mainly osteosarcoma (OS), chondrosarcoma (CS) and Ewing's sarcoma (ES). OS is the most common bone tumour 138, while ES and CS arise from bone/soft and cartilage tissues, respectively 138. Unlike other tumours, which are diagnosed in the elderly 139-142, OS and ES affect children, adolescents and young adults 143. CS is instead considered an adulthood cancer 138. Bone tumour onset/progression depends on the dysregulation of different factors, including miRNAs and targets.

The miR-34 family (miR-34a, -b, and -c) plays a role in regulating OS essential targets, including Notch, p53, Bcl-2, PI3K/Akt, c-Myc, Bim and Runx2 144. The members of this miR family present tumour suppressive characteristics, which are downregulated in OS 144. The miRNA cluster made up ofmiR-127-3p, -154, -299-5p, -329, -337-3p, -376a/c, -377, -382, -409, -410, -432, -493, -495, -453, -654-5p and -758, which is located within the chromosome 14q32 locus 145, appears to be very significant. Indeed, these miRNAs are downregulated in OS 145, while their main target is the onco-protein c-Myc. Recent studies have confirmed that miR-143 is downregulated, while miR-21, -221 and -106a are up-regulated in OS tissues. Their differential expression is also associated with tumour staging and grading, as well as lung metastasis 146. Specifically, miR-21 and -221 play a role in OS pathogenesis as oncogenic miRNAs (onco-miRs) 147. MiR-21 is involved in the control of TGF-β1 147, which regulates cell growth, differentiation and apoptosis. *PTEN* and *PDC4* are two additional targets for miR-21. This interaction leads to OS cell growth, migration, invasion and metastasis 148. Since miR-21 inhibition in patients significantly delays OS progression, this miR is considered to be a prognostic marker 148. MiR-221 over-expression induces cisplatin resistance and reduces apoptosis in OS cell lines via PTEN inhibition, while its silencing overturns these effects 149. The increased expression of miR-221 has also been associated with OS tumour staging, metastatic stating, as well as chemotherapy response 150. For these reasons, miR-221 can be employed as a marker in OS staging, metastasis and prognosis 150. Other potential OS prognostic markers include miR-15b, -378, -568, -574, -494 and -601. These miRNAs are involved in chemotherapy responses in OS patients 151. The expression of several 14q32-associated miRNAs, including miR-139-5p, -299, -323, -379, -382, -411, -493, -539 and -758, has also been linked to OS recurrence-free survival 152. Additional miRNAs, i.e.miR-134, -382 and -544 have been inversely correlated to aggressive OS behavior, increased metastatic potential and accelerated time to death 152. Circulating miRNAs which were found to be dysregulated in serum could represent OS diagnostic/prognostic tools. The plasma/serum levels of miRNA-34b have been detected as lower in OS patients and related to metastasis status 153. MiR-21 and -148a, have been found to be dysregulated in OS patients sera and/or plasma 154,155 and could therefore be used for diagnostic purposes. Circulating miRNAs could also improve OS type identification. For instance, osteoblastic OS type is associable with miR-21 and -320a increase and miR-143 and -199a-3p decrease in sera 156. In addition, miR-21 content tested high in sera from late-stage OS patients. The presence of some specific miRNAs has been associated with increased risk of both local recurrence and metastasis and worse survival rates in patients with osteosarcoma. In patients who have OS, miRNAs profiles in those with pathologic fractures are different from patients without pathologic fractures. MiR-656-3p, miR-493-5p and miR-381-3p were up-regulated in patients with pathologic fractures, whereas miR-363, miR-85-5p and miR-20b-5p were downregulated.

MiRNAs dysregulation is a critical process in CS onset/progression. Several miRNAs, including miR-30a 157 and -494 158, regulate several SOX gene family members (*SOX4*, -*5* and -*9*) which play crucial roles in CS 159. One of these miRNAs, miR-494, has also been linked to poor CS survival/prognosis, underlying that it may be used as a prognostic marker for therapy 158. An anti-tumour effect has been reported for miR-23b, which is down-expressed in CS and targets an important factor of CS pathogenesis, i.e., Src kinase 160. Interestingly, treating CS cells with (i) naringin, which is a compound belonging to the flavonoid family, and/or (ii) paeonol, other than suppressing cell migration/invasion, also changes miRNA expression 161,162. Indeed, naringin induces miR-126 over-expression, followed by cell migration and invasion inhibition via VCAM-1 down-regulation 162, while paeonol increases miR-141 levels, which in turn inhibit cell motility by PKC and c-Src kinase activities 161. Previous studies, which focused on chemokine CCL5 and VEGF-dependent angiogenesis, underlined the importance of miRNA dysregulation in CS. Indeed, CCL5 has been reported as promoting VEGF-dependent angiogenesis and negatively regulating-199a in CS cells 163. Moreover, another study indicated miR-181a as a hypoxia-regulated miRNA which controls VEGF expression in CS cells 164. Therefore, this miRNA could be considered as a potential therapeutic tool for CS angiogenesis monitoring 164. Additionally, miR-181a overexpression causes the development of ossification in the posterior longitudinal ligament (OPLL) by targeting PBX1 165. Other miRNAs, which are involved in CS onset/development are miR-125b, which tested as down-regulated in both CS tissues/cell lines 166, whereas the miR-143/145 cluster presents tumour suppressor features 167. The former, when overexpressed, inhibits cell proliferation and enhances cell sensitivity to doxorubicin (a chemotherapy agent) by targeting the oncoprotein ErbB2 166. The latter directly inhibits FSCN1, which is a malignancy-promoting protein associated with CS progression 167. Lastly, additional miRNAs whose dysregulation has been found to be involved in CS onset/development include hub-miRNAs, such as miR-622, -4539, -145, -25 and -96, which are dysregulated in CS tissues/cells 168.

ES pathogenesis is linked to miRNAs dysregulation. The EWSR1/FLI1 fusion protein, which is the main oncogenic factor in ES, negatively regulates miR-193bexpression. This miRNA also targets the oncoprotein ErbB4 to inhibit anchorage-independent growth 169. In addition, a set of additional 91 miRNAs, including miR-145, -205, -190 and -35a-5p fall under EWSR1/FLI1 regulation 170. Among these, miR-35a-5p controls cell proliferation/invasiveness in ES cells by targeting CD99, which is an ES-related oncoprotein^170^. Other miRNAs have been identified as dysregulated in ES. A group of 58 miRNAs have been identified as dysregulated in ES 171. Among these, miR-21, -181a/b, -29a/b, -497, -195, -let-7a, -34a and -1915 share *BCL-2* as the same target 171. Another study has reported the altered expression of miR-21, - 31, -106b, -145, -150, 371-5p, -557 and -598 in ES animal models. These miRNAs have been predicted to regulate a number of ES-related genes, including *IGF1*, *EWSR1*, *FLI1* and their fusion gene 172. Several miRNAs which have been identified as tumour suppressors in ES include miR-30d, -107, -124, -138 and -199b-5p 173. Moreover, miR-138 plays a role as a tumour suppressor in osteosarcoma by targeting DEC2 174. In addition, miRNAs belonging to the miR-17~92a, -106b~25 and -106a~363clusters are dysregulated in ES 175. Among these, the miR-17~92 cluster controls TGFB/BMP pathways, whose down-regulations are crucial for ES pathogenesis 175. Previous works have suggested a potentially regulatory role for miR-21 in ES pathogenesis by targeting Activated Leukocyte Cell Adhesion Molecule (*ALCAM*/*CD166*), whose expression is characteristic of tumourigenicity 176. Another piece of work has identified miR-199a-3p as a key driver which is able to reverse ES malignancy by inhibiting cell migration through the abrogation of CD99, which is a membrane protein expressed in ES 177. MiR-301 has also been reported to acts as an onco-miR in ES cells, promoting cell cycle progression and proliferation by *PTEN* inhibition 178. Lastly, miRNAs also play a role in ES metastasis. MiR-124over-expression leads to ES cell invasion *in vitro* and metastasis *in vivo* 179, whereas miR-138 inhibits the ES metastatic potential by Focal adhesion kinase (FAK) suppression 180.

The abovementioned studies suggest that a number of miRNAs are dysregulated in OS, CS and ES and thus representing potentially promising tools for the diagnosis, prognosis and treatment of these bone tumours. MiRNAs could also be employed as novel therapeutic targets for bone tumour therapy.

## MiRNA-based therapies and delivery systems

MiRNA-based therapies could be applied for both cancer treatments and regenerative medicine. The most challenging issue in the application of miRNA-based therapies is the development of safe and efficient delivery systems. To this purpose, the use of miRNA-based scaffolds could allow spatio-temporal control and avoid biological and mechanical barriers that inhibit successful miRNA delivery 181. In regenerative medicine, scaffolds act principally as templates for cell attachment and tissue repair. To date, a variety of scaffolds (e.g., collagen scaffolds, solid porous scaffolds and hydrogel scaffolds) have been developed to locally deliver miRNAs to cells at the site of damage 182. Therefore, the use of scaffold-based miRNAs to deliver therapeutics could be a better method to locally release miRNAs while prolonging the delivery time-frames 183. MiRNA-based therapy applies both cell-free and cell-mediated scaffolds, also termed *in situ* or ex situ delivery, respectively. In cell-free scaffolds, miRNAs are directly loaded into the scaffold structure which are implanted in the defect site where miRNAs then interact with the resident and homing cells to regenerate such defects 181. Contrariwise, in cell-mediated scaffolds, cells are transfected with miRNAs *in vitro*, loaded onto scaffolds, and finally implanted into the damage site 181. Ultimately, a series of issues still need to be resolved before miRNA scaffold-based therapies can be used, including developing a more efficient scaffold-vector delivery system, improving biological stability, increasing specific targeting capacity, and prolonging presentation in addition to reducing off-target effects 182. Several reports for scaffold-based miRNA delivery fields have focused on the application of miRNA 26a in inducing bone regeneration both in cell-mediated and cell-free scaffolding 184-187. While, in the cartilage regeneration field, the most interesting study describing scaffold-based miRNA delivery has reported on a new strategy based on hydrogel-mediated delivery of an antimiR-221 to induce cartilage repair 188.

## Conclusion

MiRNAs play a pivotal role in cellular function, including MSC survivability and differentiation. Several studies have been performed to better characterise miRNAs and their unique regulatory functions. This review has highlighted that these molecules can exert different effects on chondrogenic and osteogenic differentiation. As discussed above, some miRNAs can exhibit contrasting effects on chondrogenesis and osteogenesis while others can exert both stimulatory and inhibitory effects. MiRNAs are also involved in the pathogenesis of diseases related to cartilage and bone tissue. Indeed, some miRNAs, such as miR-21, have been found to be dysregulated in OP, ES and OS patients. Serum miRNA, miR-140-3p, alongside miR-23b-3p, could be considered potential biomarkers for OP and OP fractures in post-menopausal women. Several studies have demonstrated miR-148a dysregulation in bone fractures, OP and OS patients. Thus, it represents a diagnostic marker for such pathologies. MiR-494, which is related to poor prognosis in this pathology, could be a promising diagnostic marker for CS. Therefore, miRNAs could be employed as novel therapeutic targets for bone and cartilage disease/tumour therapy. The identification/validation of these miRNAs and their targets, in association with further research into new safe, efficient delivery systems for miRNAs or their regulation tools, could encourage the transfer of miRNAs into clinical practice.

## Future perspectives

The role of miRNAs as regulators of MSC chondrogenic and osteogenic differentiation and their involvement in cartilage and bone disease has been largely described in this review. The studies cited herein have described the most well-known function of miRNAs, which is to negatively regulate gene expression by interfering with mRNA. One strategy to improve the chondrogenic and osteogenic potential of MSCs could be to either expose cells to mimic/antago-miRs that enhance or inhibit the effect of these miRNAs, respectively. MiRNA-based therapy applies both cell-free and cell-mediated scaffolds, which is also termed *in situ* or ex situ delivery, respectively. In cell-free scaffolds, miRNAs are directly loaded into the scaffold structure and are implanted into the defective site where miRNAs then interact with the resident and homing cells to regenerate damaged tissue. Contrariwise, in cell-mediated scaffolds, cells are transfected with miRNAs *in vitro*, loaded onto scaffolds, and finally implanted into the damaged site. *In vivo*, interaction between transplanted MSCs, neighboring cells, and humoral factors secreted from the neighboring cells is believed to influence the proliferation and differentiation of MSCs via miRNAs. In this context, human gingival fibroblasts (HGF), a type of periodontal cell, are believed to influence osteogenesis in hMSCs. Indeed, HGF secrete Insulin-like growth factor (IGF)-1 which regulates miRNA expression. Specifically, miR-101-3p and the transcription factor Ets variant 1 (ETV1) are key factors which are regulated by IGF-1 189. In the same way, it has been reported that humoral factors from human periodontal ligament cells (HPL cells) co-cultured with hMSCs suppress osteogenesis in hMSCs. The suppressive effect in these cells appears to be mediated by miR-299-5p and SOX11 190. However, further studies are needed to elucidate transplanted MSC site-specific differentiation.

Recently, an unconventional aspect of miRNA is emerging within the framework of complex regulatory pathogenesis networks. Specifically, some evidence highlights the role of miRNAs in controlling the expression of other miRNAs in a mechanism known as miRNA-miRNA interaction. MiRNA regulation by another miRNA mainly depends on nucleotide complementary sequencing. Interaction may occur between miRNA and pri-miRNA or mature miRNA, or indirectly by targeting TFs or miRNA regulators. MiRNA-miRNA crosstalk has been reported as being involved in the onset of different diseases, such as cardiac pathologies, ovarian cancer and head and neck squamous cell carcinoma (HNSCC). MiR-21 has been found to be up-regulated in many solid human malignancies, whereas it participates in several miRNA-miRNA interactions. Specifically, miR-21 interacts with miR-499 and miR-145 leading to HNSCC invasion and colorectal cancer, respectively 191, while interaction between miR-21 and miR-122 induces hepatocellular carcinoma 192. These discoveries, in combination with those reported in this review, indicate that crosstalk miRNA-miRNA deserves to be explored in order to understand the complex hidden networks that cause cartilage and bone diseases. Another miRNAs mechanism of function, which deserves to be looked into with future research, is the crosstalk between miRNAs and other non-coding RNA molecules such as long non-coding RNAs (lncRNAs). It has been reported that interplay between lncRNAs and miRNAs is involved in MSCs osteogenic differentiation 193. Consequently, new studies into crosstalk between lncRNAs-miRNAs in cartilage and bone diseases would be relevant.

Although much work still needs to be done, the development of efficient miRNA-based therapies would appear to offer significant promise, particularly in personalized medicine.

## Figures and Tables

**Table 1:** MicroRNAs involved in chondrogenic differentiation, their direct target genes and cell type in which the role of miRNAs has been studied

miRNA	Cell type	Target gene	Effect on chondrogenesis
miR-20a	ATDC5	Atg7	-
miR-20b	iPSC	EPAS1	-
miR-29a	MSCs, synovial fibroblasts	FOXO3A, VEGF	-
miR-520d-5p	hMSCs	HDAC1	+
miR-381	ATDC5, SW1353	HDAC4	-
miR-1	Primary chicken chondrocyte	HDAC4	-
miR-221	MSCs	FOXO3	-
miR-27b	NP cells	MMP-13	+
miR-9	Chondrogenic progenitors, articular chondrocytes	MMP-13, PRTG	+
miR-124	murine BM-MSCs	NFATC1	-
miR-92a	Chondrogenic progenitors	Nog3	+
miR-127-5p	Rat BM-MSCs	MMP-13	+
miR-182-5p	BM-MSCs	PTHLH	-
miR-140-5p	hMSCs	RALA	+
miR-455-3p	ATDC5	RUNX2	+
miR-320	ATDC5	RUNX2, MMP13	+
miR-30a	hMSCs	SOX9	-
miR-30b	C3H10T1/2	SOX9	-
miR-574-3p	hMSCs	RXRα	+
miR-199a	C3H10T1/2	SMAD1	-
miR-155	A549	SMAD1	+
miR-194	MSCs	SOX9 or SOX5	-
miR-145	murine and human MSCs	SOX9	-
miR-101	Rat BM-MSCs	SOX9	-
miR-1247	human chondrocytes	SOX9	-
miR-495	MSCs	SOX9 or SOX5	-
miR-140	hMSCs, mouse cells	HDAC4, ADAMTS‐5	-
miR-337	human and rat chondrocyte	TGFBR2	-
miR-193b	ATDC5, hASCs	TGFBR2, DDAH1	-
miR-17-92 cluster	MPCs	TGF-β signaling pathway, SQSTM1/p62	-
miR-210	Rat Medial Meniscal Model	HOXA-1/9	+
miR-107	C28/I2 cell	PTEN	+
miR-424	hiPSCs	VEGF, VEGFR2, FGFR1	+

**Table 2:** MicroRNAs involved in osteogenic differentiation, their direct target genes and cell type in which the role of miRNAs has been studied

miRNA	Cell type	Target gene	Effect on osteogenesis
miR-98	hMSCs	BMP-2	-
miR-140‐5p	hMSCs	BMP-2	-
miR-370	MC3T3‐E1	BMP-2	-
miR-93-5p	hMSCs	BMP-2, SP7	-
miR-195-5p	PDLSCs	BMPR1A	-
miR-100	hMSCs	BMPR2	-
miR‐106a‐5p	PDLSCs	BMP-2, E2F5	-
miR-153	hMSCs	BMPR2	-
miR-132	UC-MSCs	β-catenin	-
miR-29a	BM‐MSCs, MC3TC‐E1	HDAC4	+
miR-29b	BM‐MSCs, MC3TC‐E1, USSC	HDAC4	+
MiR-376c-3p	BM-MSCs	IGF1R	-
miR-410-3p	hRIFs	MSX2	-
miR-23a cluster	MC3T3‐E1	Prdm16	+
miR-140	hPDLFs	RhoA	-
miR-23a	MSCs, MC3T3-E1	RUNX2	-
miR-23b	MSCs	RUNX2	-
miR-30b	MSCs	RUNX2	-
miR-30c	MSCs	RUNX2	-
miR-133a-5p	MC3T3-E1	RUNX2	-
miR-135a-5p	C2C12	RUNX2	-
miR-137	MSCs	RUNX2	-
miR-137-3p	BM-MSCs	RUNX2	-
miR-203	MSCs, MC3T3‐E1	RUNX2, MAPK/STAT pathways activation	-/+
miR-204	MSCs, MPCs, BM-MSCs	RUNX2	-
miR-211	MPCs, BM-MSCs	RUNX2	-
miR-217	MSCs	RUNX2	-
miR-221	MSCs	RUNX2	-
miR-338	MSCs	RUNX2	-
miR-433	C3H10T1/2	RUNX2	-
miR-505	MC3T3-E1	RUNX2	-
miR-628-3p	MG63	RUNX2	-
miR-1305	PDLSCs	RUNX2	-
miR-30 family	MC3T3-E1, MSCs	RUNX2, SMAD1	-
miR-338-3p	BM-MSCs, MC3T3-E1	RUNX2, FGFR2	-
miR-205	MSCs, BM-MSCs	RUNX2, SATB2	-
miR-222-3p	BM-MSCs	RUNX2, SMAD5	-
miR-133a	MSCs, C2C12	RUNX2, SMAD5	-
miR-135a	MSCs, MC3T3-E1, ATDC5	RUNX2, SMAD5	-
miR-143	MSCs	RUNX2, SP7	-
miR-34c	MSCs, MC3T3‐E1	RUNX2	-
miR-375	C2C12, hASCs	RUNX2, Yap1	-/+
miR-133	C2C12	RUNX2	-
miR-130a-3p	hASCs	SIRT7	+
miR-26b	USSC	HDAC4	-
miR-203-3p	MSCs	SMAD1	-
miR-26a	USSC, BM-MSCs, hASCs	SMAD1, GSK3b, HDAC4	-/+
miR-135	C2C12	SMAD1, SMAD5	-
miR-146a	ADSCs	SMAD4	-
miR-144-3p	C3H10T1/2	SMAD4	-
miR-17-5p	BM‐MSCs	SMAD7	+
miR-21-5p	BM-MSCs	SMAD7	+
miR-21	BM-MSCs	SMAD7	+
miR-21a	C3H10T1/2	SMAD7	+
miR-590-5p	C3H10T1/2, MG63, MC3T3-E1	SMAD7	+
miR-497-5p	SCAP	Smurf2	+
miR-93	C57BL/6	SP7	-
miR-637	hMSCs	SP7	-
miR-145	C2C12, MC3T3-E1, BM-MSCs	SP7, CBFB	-
miR-214	BM‐MSCs, MC3T3‐E1, C2C12, C57BL/6	SP7, ATF4, β-catenin, FGFR1	-
miR-378	hMSCs	Wnt6, Wnt10	-
